# Pseudoprogression in Koos grade 4 vestibular schwannomas following stereotactic radiosurgery: temporal dynamics and radiological predictors

**DOI:** 10.1007/s11060-026-05619-y

**Published:** 2026-05-25

**Authors:** Sammy M. Schouten, Stefan Cornelissen, Patrick P. J. H. Langenhuizen, Henricus P. M. Kunst, Jeroen B. Verheul

**Affiliations:** 1 Department of Neurosurgery, Gamma Knife Center, Elisabeth-TweeSteden Hospital, Hilvarenbeekseweg 60, Tilburg, 5022 GC The Netherlands; 2 Department of Otolaryngology, Radboud University Medical Center, Nijmegen, The Netherlands; 3 Department of Otolaryngology, Maastricht University Medical Center+, Maastricht, The Netherlands; 4 Dutch Academic Alliance Skull Base Pathology, Nijmegen & Maastricht, The Netherlands; 5 Architectures for Reliable Imaging Analysis Group, Eindhoven University of Technology, Eindhoven, The Netherlands

**Keywords:** Koos grade 4, Pseudoprogression, Stereotactic radiosurgery, Gamma Knife, Vestibular schwannomas, Transient tumor enlargement.

## Abstract

**Purpose:**

To gain insight into the volumetric temporal response dynamics following primary stereotactic radiosurgery (SRS) and to evaluate possible MRI-derived predictors at time of treatment for pseudoprogression in sporadic Koos grade 4 vestibular schwannomas (VS).

**Methods:**

Patients treated with Gamma Knife radiosurgery for a unilateral sporadic Koos grade 4 VS, larger than 4 cm^3^ at time of treatment, were included. In tumors with cystic components, separate annotations of the solid and cystic components were additionally obtained. Uni- and multivariable logistic analyses were performed to evaluate possible predictors for pseudoprogression.

**Results:**

A total of 258 patients with a median tumor volume of 6.8 cm^3^ (IQR 5.1–8.9) were included in the study. Pseudoprogression was observed in 75 tumors (29%) with a median time-to-peak of 8 months (IQR 6–14) and a median relative increase of 23% (IQR 15–34) at peak. In 19 out of 39 macrocystic tumors, the solid versus cystic components exhibited distinct response dynamics. Multivariable logistic regression analyses revealed that only the presence of microcystic components was significantly inversely associated with pseudoprogression with an adjusted OR of 0.15 (95% CI 0.05–0.46; *P* < 0.001). Ultimately, 30 patients (12%) required 31 additional interventions after a median of 36 months (IQR 21–60).

**Conclusions:**

Pseudoprogression rates in selected Koos grade 4 VS appear comparable to those of smaller tumors. Tumors with microcystic components exhibited significantly lower pseudoprogression rates, which may render them more suitable candidates for SRS. In contrast, macrocystic tumors demonstrated more variable dynamics, warranting careful monitoring in these tumors.

**Supplementary Information:**

The online version contains supplementary material available at 10.1007/s11060-026-05619-y.

## Introduction

Vestibular schwannomas (VS) are benign intracranial tumors originating from the eighth cranial nerve and are the most common cerebellopontine angle neoplasm. Active treatment strategies include stereotactic radiosurgery (SRS), microsurgery, or a combination of these methods, aiming to prevent mass effect complications in neighboring neurovascular structures due to tumor growth or large tumor size [1–3]. In small- to medium-sized VS, the less invasive SRS is a commonly used treatment option with high neurofunctional outcome rates and successful tumor response rates of > 90% [4]. Conversely, for large VS, commonly classified as Koos grade 4, there remains a global discrepancy in the preferred treatment modality. Most centers and guidelines opt for microsurgery to ensure long-term tumor control and to avoid risk of radiation-induced mass effect complications, mainly caused by pseudoprogression [3, 5, 6]. However, SRS, delivered as either single-session or hypofractionated treatment, has recently gained popularity and evidence as an alternative, safe, and effective treatment for selected large VS [4, 7–9]. Especially for minimally symptomatic patients with a good neurofunctional status or those unsuitable for invasive surgery, given the associated surgical risks that significantly escalate with tumor size [4, 7–9].

Pseudoprogression, characterized by transient tumor enlargement subsequent to radiation exposure, is observed in a considerable proportion of VS, mainly within the initial 3 years post-SRS, with reported incidences between 18% and 72% [10–21]. Particularly in larger VS, the potential occurrence of pseudoprogression may influence clinical decision-making. Given the limited space available, expansion can lead to the progression of mass-effect complications, necessitating surgical intervention. To date, there are still no reliable predictors for pseudoprogression, and most prior studies involve smaller tumors or limited series [10–22]. A clearer understanding of pseudoprogression in larger VS could improve individualized counseling and treatment decision-making in these selected patients.

The aim of the current study was to assess the volumetric response dynamics in sporadic Koos grade 4 VS following primary SRS and to evaluate possible MRI-derived predictors for pseudoprogression.

## Methods

### Study population

For this retrospective study, institutional review board approval was obtained and the requirement for informed consent was waived by the regional medical ethics committee. Patients treated with Gamma Knife radiosurgery (GKRS) between January 2004 and January 2021 for a unilateral sporadic Koos grade 4 VS, larger than 4 cm^3^ at time of treatment, were identified. The exclusion criteria were as follows: previous unilateral cranial surgery or radiation therapy, evidence of neurofibromatosis type 2, and less than two years follow-up post-GKRS. Patients converted to second treatment within the two years post-GKRS due to tumor expansion were included.

Baseline patient demographic data and presenting symptomatology at time of treatment were collected from institutional electronic medical records. Tumor-specific characteristics were obtained by reviewing the MR images and radiological reports. Presence of peritumoral edema was assessed on T2-weighted imaging. Presence of microcystic components was defined as having one or more small- to moderate intratumoral cysts and presence of macrocystic components was defined as having large peri- and/or intratumoral cysts compromising more than 50% of the total tumor volume [23]. See Supplemental Figure S1 for examples of typical tumors with microcystic components versus macrocystic components. Degree of tumor middle cerebellar peduncle (MCP) compression was quantified by measuring the absolute and relative difference of the MCP width on the tumor side versus the contralateral side in the axial plane, as measured in Hasegawa et al. [24].

### Treatment and follow-up

At our center, GKRS is offered as a treatment option for Koos grade 4 VS smaller than 15 cm^3^ (approximately 30 mm maximum extrameatal diameter) without clinically relevant mass effect (e.g., hydrocephalus or ataxia). The distinction is made on a case-by-case basis within the multidisciplinary skull-base team, taking into account both imaging findings and clinical presentation. In exceptional circumstances, larger tumors (up to 20 cm^3^) were also treated when patients were deemed medically unfit for surgery [7, 25]. GKRS was performed in a single fraction with frame-based fixation using the Leksell Gamma Knife (Elekta AB, Stockholm, Sweden) model 4 C and since 2008, model Perfexion. A 13 Gy dose was prescribed to the isodose line, covering 90% (until May 2011) to 99% (since May 2011) of the tumor volume, ensuring a marginal dose (99% coverage) of at least 11 Gy. Treatment planning was based on pre- and post-contrast T1-weighted (T1CE) and T2-weighted MRI sequences. Following treatment, patients were scheduled for biannual follow-up during the first year and annual follow-ups thereafter. At each follow-up, at least a T1CE MRI with 1 mm slice thickness was conducted. Standard follow-up intervals were shortened in cases of suspected radiological progression or progression of symptoms, and intervals were extended in cases of sustained radiological regression or stability.

### Volumetric analysis and outcome

Tumor measurements were conducted semi-automatically slice-by-slice on the T1CE scans at treatment and follow-up using GammaPlan (version 11.3, Elekta AB, Stockholm, Sweden). In growing tumors prior to GKRS treatment, the pretreatment volume was additionally determined using the oldest available MRI scan obtained more than 6 months before treatment. The pretreatment growth rate was subsequently calculated following the volume doubling time (VDT) model proposed by Varughese et al. [26].

In tumors with cystic components, separate annotations of the solid and cystic components were additionally obtained. Annotation was performed by the authors (S.S., S.C., P.L., and J.V.), each possessing 3–21 years of experience in volumetric segmentation of VS. An inter-observer variability study involving the same annotators demonstrated excellent agreement among annotators, with an interrater correlation coefficient of 0.995 [27]. Based on previous evidence, a significant volume increase within 4 years post-SRS followed by significant regression was defined as pseudoprogression [10, 28, 29]. A significant change in tumor volume was defined as a longitudinal relative change of ≥ 10% [16, 20, 27, 30, 31].

### Statistical analysis

Associations of baseline patient- and tumor characteristics at time of treatment with occurrence of pseudoprogression were evaluated using uni- and multivariable logistic regression analyses and summarized with odds ratios (ORs) and 95% confidence intervals (CIs). Variables with a nonlinear association with the log odds probability for pseudoprogression were categorized into quartiles. Multivariable analyses were only performed for age, tumor size, and variables with a significant association with pseudoprogression in univariable analysis. The OR in multivariable analysis is adjusted for age, tumor size, presence of cystic components, presence of peritumoral edema, and the prescribed marginal dose in Gy. Additionally, in several cases, pseudoprogression status could not be determined either because of (1) unavailable 3–9 months follow-up imaging in tumors that subsequently regressed or remained stable, or (2) additional treatment within two years post-GKRS. These undetermined cases were excluded from logistic analyses. A separate sensitivity analysis was performed to evaluate the impact of this approach and is provided in the Supplementary Results S4. The association of pretreatment growth rate with pseudoprogression was assessed separately for growing tumors prior to GKRS. Intervention-free survival rates were estimated using the Kaplan–Meier method. A *P*-value of < 0.05 was considered significant. Statistical analyses were performed with SPSS Statistics for Windows, Version 29 (IBM Corporation, Armonk, NY, USA).

## Results

### Study population

A total of 269 patients were treated with primary GKRS for a unilateral sporadic Koos grade 4 VS (> 4 cm^3^) between January 2004 and January 2021. Ultimately, 258 patients could be included (See flow diagram study: Supplementary Figure S2). The median age at treatment was 58 years old (IQR 47–67) and the median tumor size at treatment was 6.8 cm^3^ (IQR 5.1-9.0). Further patient- and tumor characteristics at time of treatment are summarized in Table 1.

**Table 1:** Summary of patient- and tumor characteristics at baseline and follow-up data^a^

Characteristic	Total *N* = 258	Missing *N*
Age at time of treatment in years	58 (47–67)	0
Male gender	128 (50)	0
Treatment indication		
Size	139 (54)	
Growth	109 (42)	
Hearing preservation	6 (2)	
Patient preference	1 (0.4)	
Unspecified	3 (1)	
VDT in months of growing tumors prior to GKRS	17 (10–35)	21
Prescription dose in Gy	13.0 (12.5–13.0)	0
Isodose line in %	50 (45–59)	0
Marginal dose (99% coverage) in Gy	12.3 (11.6–12.9)	0
Dose to 90% tumor volume in Gy	13.8 (13.0-14.4)	0
Maximum dose in Gy	25.5 (21.7–28.3)	0
Serviceable hearing ^*b*^	106 (45)	23
Tinnitus	159 (67)	22
Dizziness	145 (62)	23
Trigeminal dysfunction ^*c*^	79 (32)	13
Facial numbness	68 (28)	
Facial pain	15 (6)	
Facial paresis	3 (1)	0
HB 2	2 (1)	
HB 3	0 (0)	
HB 4	1 (0.4)	
HB 5	0 (0)	
HB 6	0 (0)	
Cerebellar symptoms	10 (4)	0
Tumor volume in cm^3^	6.8 (5.1-9.0)	0
Maximum extrameatal diameter in mm	26 (23–30)	0
Cystic components	81 (31)	0
Microcystic	42 (16)	
Macrocystic	39 (15)	
Peritumoral edema	107 (46)	25
Absolute MCP compression in mm	8 (6–9)	0
Relative MCP compression in %	47 (39–54)	0
Number of follow-up MRIs ^*d*^	6 (4–7)	0
Conversion to second intervention ^*e*^	30 (12)	N/A
Second GKRS treatment	12	
Drain	6	
Microsurgery	13	
Time to second intervention in months	36 (21–60)	N/A
Follow-up of non-intervened tumors in months	84 (48–122)	N/A

^a^ Summarized with median (IQR) or n (% of total). ^b^ Gardner-Robertson score 1 or 2. ^c^ Six patients had both facial numbness as facial pain. ^d^ Excluding treatment MRI. ^e^ One patient received a drain and subsequently underwent microsurgery. Abbreviation: VDT, Volume Doubling Time; HB, House-Brackmann facial paralysis scale; GKRS, Gamma Knife Radiosurgery

### Temporal dynamics of pseudoprogression

Tumor enlargement following GKRS was observed in 103 tumors (40% of total cohort), with 94 occurring within 4 years. Of these, 75 tumors (29% of the total cohort) demonstrated transient enlargement attributed to pseudoprogression, 9 tumors (3% of the total cohort) showed continuous enlargement, and one tumor (0.4%) initially regressed post-GKRS before exhibiting continuous enlargement. For the remaining 9 tumors (3%), the growth dynamics remained undetermined due to subsequent treatment within two years post-GKRS.

**Fig. 1:**
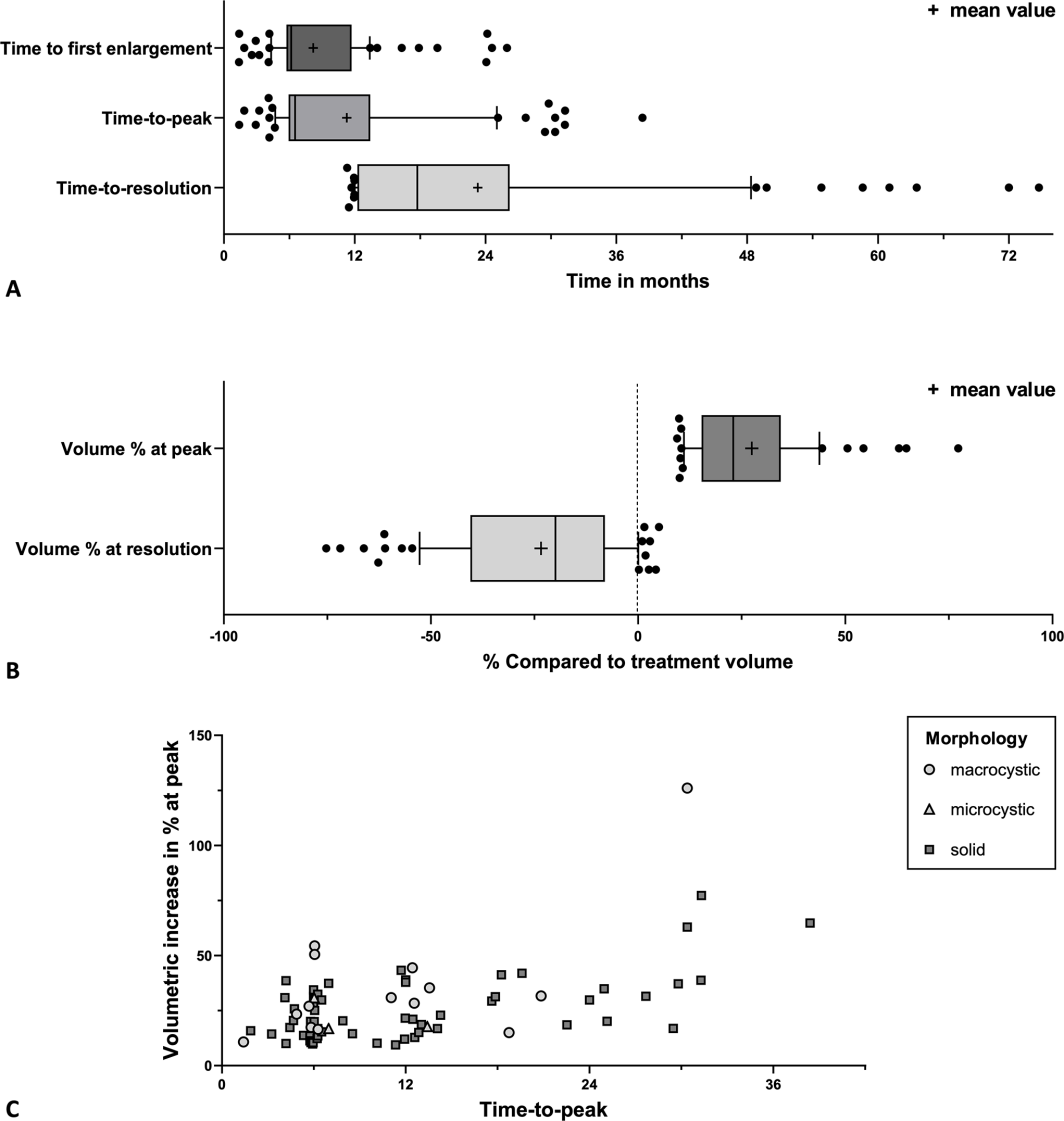
Pseudoprogression characteristics of this cohort (*N* = 75): (**A**) Summarizing boxplots with 10–90 percentile whiskers and mean value (+) of time to first enlargement, time-to-peak, and time-to-resolution of pseudoprogression; (**B**) Summarizing boxplots with 10–90 percentile whiskers and mean value (+) of tumor volume percentage compared to treatment volume at peak and at time of observed pseudoprogression resolution; (**C**) Scatterplot correlating individual tumors between time-to-peak and the extent volumetric increase in % at peak

Among the 75 tumors demonstrating pseudoprogression, the median and mean time-to-peak were 8 months (IQR 6–14; range 2–38) and 11 months (Fig. 1A). The median and mean volumetric increase at peak were 23% (IQR 15–34) and 27% (Fig. 1B). Fifty cases (67% of the pseudoprogression cases) had an increase of more than 20%. The correlation between time-to-peak and the extent of relative increase at peak in % for all individual pseudoprogression cases are presented in Fig. 1C. One outlier, characterized by macrocystic components, exhibited a volumetric relative increase of 126% at 30 months post-GKRS. The median and mean time-to-resolution observed is 18 months (IQR 12–27) and 25 months, with 90% of the pseudoprogression cases resolving within 4 years (Fig. 1A). See Supplemental Figure S3 for representative pseudoprogression cases on MRI at time of treatment and during follow-up.

In 42 tumors (16% of the total cohort), the occurrence of pseudoprogression remained undetermined: 9 tumors (3%), as aforementioned, due to subsequent intervention within two years post-GKRS following observed enlargement, and 33 tumors (13%) due to continuous regression or stability without an available follow-up scan at 3–9 months post-GKRS. Consequently, the estimated pseudoprogression rate in this cohort ranged from 29% to 45% (Supplementary Results S4).

### Uni- and multivariable analyses for pseudoprogression

In uni- and multivariable analyses, only the presence of microcystic tumor components was significantly inversely associated with pseudoprogression, yielding a highly significant adjusted OR of 0.15 (95% CI 0.05–0.46; *P *< 0.001) compared to non-cystic tumors (Table 2). In the secondary analysis of growing tumors (*N* = 109) prior to GKRS, pretreatment growth rate was known for 88 tumors (81%) with a median VDT of 17 months (IQR 10–35). Of these, the pretreatment growth rate was not found to be significantly associated with the occurrence of pseudoprogression (*P* = 0.41), remaining insignificant after adjusting for cystic components and age.

**Table 2:** Uni- and multivariable logistic analyses of age and MRI characteristics with pseudoprogression

Variables ^a^		Univariable		Multivariable^b^	
		OR (95% CI)	*P*-Value	aOR (95% CI) ^c^	*P*-Value
Age in years		Overall	0.07	Overall	0.15
	18–47	(ref)	N/A	(ref)	N/A
	48–58	1.52 (0.75–3.07)	0.24	1.33 (0.64–2.80)	0.44
	58–64	0.98 (45-2.14)	0.95	1.08 (0.47–2.50)	0.86
	65–85	0.60 (0.30–1.21)	0.16	0.56 (0.26–1.23)	0.15
Tumor size in cm^3^		Overall	0.13	Overall	0.19
	4.0-5.1	(ref)	N/A	(ref)	N/A
	5.1–6.8	1.30 (0.62–2.72)	0.56	1.18 (0.54–2.56)	0.68
	6.8-9.0	1.13 (0.54–2.39)	0.78	0.99 (0.44–2.22)	0.52
	9.0-30.2	2.23 (0.98–4.60)	0.06	2.11 (0.95–4.67)	0.07
Cystic components		Overall	0.002*	Overall	**0**.002*
	none	(ref)	N/A	(ref)	N/A
	microcystic	0.15 (0.05–0.44)	< 0.001*	0.15 (0.05–0.46)	< **0**.001*
	macrocystic	1.10 (0.55–2.22)	0.82	1.26 (0.56–2.82)	0.58
Peritumoral edema		1.0 (0.99–1.01)	0.94		
Fourth ventricle deformation		1.23 (0.57–2.65)	0.60		
Absolute MCP compression in mm		Overall	0.81		
	1.1–6.4	(ref)	N/A		
	6.5–7.7	1.24 (0.59–2.57)	0.57		
	7.8–9.2	1.17 (0.57–2.41)	0.67		
	9.3–16.1	1.43 (0.70–2.95)	0.33		
Relative MCP compression in %		Overall	0.64		
	7.0–39	(ref)	N/A		
	39–47	1.21 (0.58–2.51)	0.62		
	47–54	1.29 (0.62–2.67)	0.50		
	54–86	1.61 (0.78–3.31)	0.20		
Marginal dose in Gy		Overall	0.38		
	10.9–11.5	(ref)	N/A		
	11.6–12.3	0.94 (0.39–2.28)	0.89		
	12.3–12.8	1.01 (0.41–2.45)	0.99		
	12.9–13.7	0.52 (0.21–1.28)	0.16		
Maximum dose in Gy		Overall	0.59		
	17.9–21.7	(ref)	N/A		
	21.7–25.5	1.29 (0.56–2.98)	0.55		
	25.5–28.2	0.79 (0.34–1.83)	0.58		
	28.3–36.5	1.24 (0.55–2.79)	0.60		

^a^ All continuous variables were categorized in quartiles due to all a non-linear relationship with the outcome pseudoprogression, range of values reported per quartile. ^b^ Multivariable analyses were only performed for significant associations with pseudoprogression (marked in bold text) in univariable analyses and tumor size. aOR is adjusted for: age, tumor size, cystic components, peritumoral edema, and marginal dose. * Significant P-value < 0.05. Abbreviation: aOR, adjusted odds ratio; MCP, Middle Cerebellar Peduncle; Gy, Gray

### Pseudoprogression in cystic tumors

Eighty-one tumors (31% of total cohort) had cystic components: 42 microcystic and 39 macrocystic. Pseudoprogression occured in 17 of 81 cystic tumors (21%), of which only 3 microcystic and 14 macrocystic. Notably, among the 14 macrocystic tumors with pseudoprogression, 10 tumors displayed discordant behaviors between their solid and cystic components. Specifically, 8 tumors showed transient enlargement only in the solid components, while 2 tumors exhibited transient enlargement solely in the cystic components.

The remaining 64 of 81 cystic tumors (79%) either showed no evidence of pseudoprogression (*N* = 56) or had an undetermined pseudoprogression status (*N* = 8). However, when the solid and cystic components were assessed separately, additional discordant behaviors emerged. Pseudoprogression occurred solely in the solid components of two macrocystic tumors and solely in the cystic components of four tumors (of which three macrocystic and one microcystic). In four macrocystic tumors, the cystic components continued to grow despite regression of the solid tumor components, necessitating surgical intervention in three cases.

### Long-term outcome

Ultimately, 30 patients (12%) required 31 additional interventions after a median of 36 months (IQR 21–60) (Table 1). In up to 7 cases, additional intervention may have been related to pseudoprogression, of these only one case was considered certain. In retrospect, intervention may have been premature in 3 patients. See Supplemental Results S4 for further details.

**Fig. 2:**
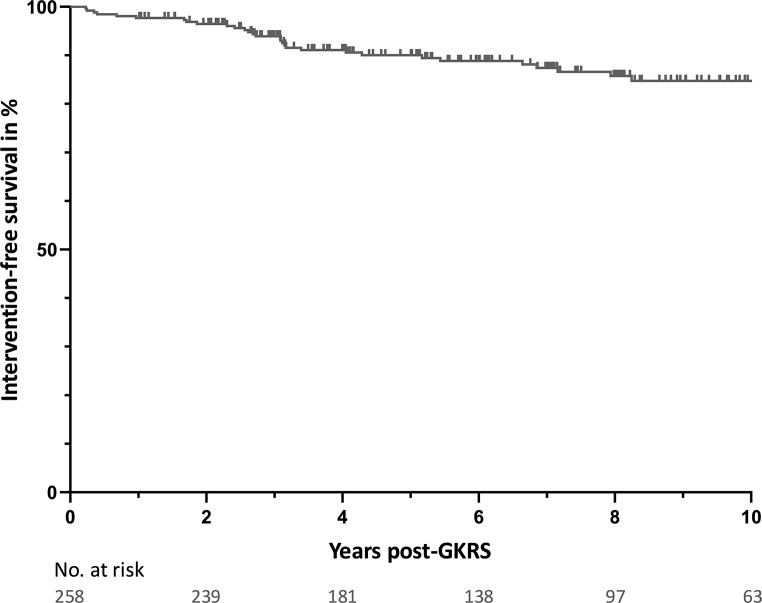
Kaplan-Meier survival curve of intervention-free survival rates post-GKRS. Abbreviation: GKRS, Gamma Knife Radiosurgery

**Fig. 3:**
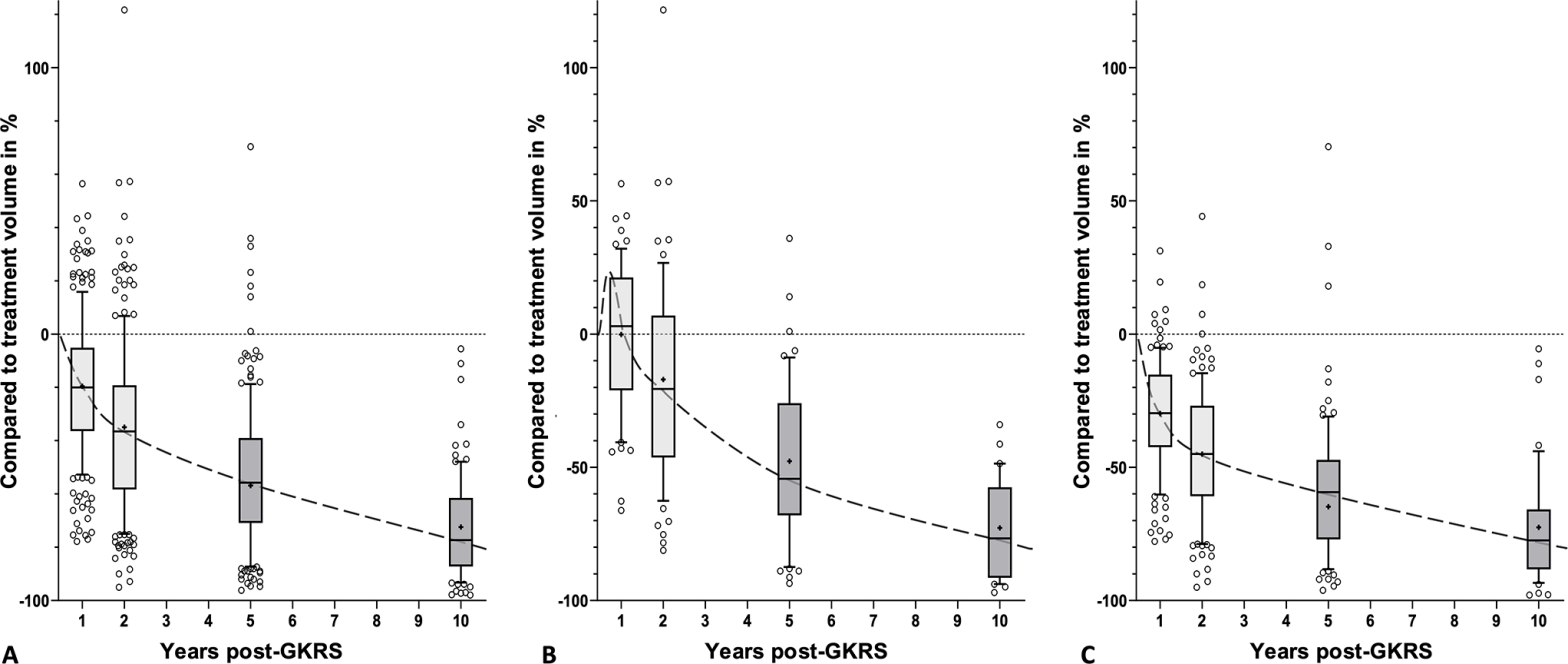
Boxplots with 10–90 percentile whiskers illustrating the volumetric change relative to tumor volume at time of treatment at 1, 2, 5, and 10 years post-GKRS. The smoothed dashed interpolated line represents the temporal dynamics of the median cohort. Results are shown for: (**A**) All non-intervened tumors, (**B**) Tumors with pseudoprogression, and (**C**) Tumors without pseudoprogression. Abbreviation: GKRS, Gamma Knife Radiosurgery

The intervention-free survival (95% CI; numbers still at risk) at 1, 2, 5, and 10 years were respectively 98% (97–99; 252), 97% (95–98; 239), 90% (88–92; 162), and 85% (82–87; 62) (Fig. 2). The median follow-up duration for the 228 non-intervened tumors (88%) was 84 months (IQR 48–121). Compared to treatment tumor volume, the median volumetric reduction at 5 years (*N* = 167) and 10 years (*N* = 83) was 57% (IQR 36–76) and 77% (IQR 61–87) (Fig. 3A). Comparison of median volumetric changes of tumors with versus without pseudoprogression (independent two-sided t-test, log-transformed due to right-skewed distribution) showed a marginal difference at 5 years (*P* = 0.05), but no significant difference at 10 years post-GKRS (*P =* 0.91) (Fig. 3B and C). Both groups had a similar available follow-up distribution.

## Discussion

Pseudoprogression is a common phenomenon in VS following SRS. In larger tumors, the occurrence may influence clinical decision-making, where tumor expansion can lead to mass-effect complications, potentially necessitating surgical intervention. The decision to intervene in cases of symptomatic pseudoprogression ultimately depends on the nature of the symptoms, the patient’s overall condition, and the clinical judgment of the treatment team. Understanding the temporal and volumetric characteristics of pseudoprogression is important to prevent premature interventions. In retrospect, intervention in this cohort may have been premature in 3 patients (Supplementary Results S4). Identifying predictors for pseudoprogression may also aid in treatment selection, particularly in borderline SRS-eligible Koos grade 4 cases with a low symptomatic profile or high surgical risk. However, robust data on pseudoprogression for large VS is scarce, with most studies focusing on smaller tumors [10–22]. This volumetric study therefore focused on Koos grade 4 VS, including 258 tumors with a median size of 6.8 cm^3^. Pseudoprogression was observed in 29% of the tumors, with an additional 16% uncertain, indicating a true incidence between 29 and 45%, consistent with prior reports on smaller VS, ranging between 19% and 72% (IQR 37–55) [10–22].

The median time-to-peak for pseudoprogression was 8 months, with a median relative increase of 23% at peak. A bimodal distribution of early versus late peak was observed, consistent with prior studies [10, 32]. Peaks ranged up to 38 months, though most cases (90%) peaked within two years post-GKRS, typically showing less than 50% volume increase. Comparison of these temporal dynamics with other studies in smaller VS revealed similar ranges, with median time-to-peak ranging from 5 to 18 months and mean maximum volume increases from 20% to 64% [10, 11, 14–19, 22]. Also, when evaluating the time-to-resolution, 90% resolved within 4 years in our cohort, comparable to other reports showing 90% resolved within 4 to 6.9 years (Fig. 2) [10, 14, 29]. Overall, these findings indicate that larger tumors respond to SRS similarly to smaller tumors.

Long-term volumetric analysis with a median follow-up of 84 months revealed substantial shrinkage in the non-intervened tumors with a median volume reduction of 77% at 10 years, regardless of pseudoprogression (Fig. 3). Previous studies likewise report that pseudoprogression does not affect long-term volumetric outcomes [16, 28, 33]. Balossier et al. [28] identified distinct post-GKRS volumetric patterns and linked pseudoprogression-related patterns to favorable outcomes. This may justify continued close surveillance of larger tumors during the initial years following treatment, even in the presence of pseudoprogression, provided no relevant mass-effect complications arise.

Logistic regression analyses revealed that only the presence of microcystic components was significantly inversely associated with pseudoprogression, with an adjusted OR of 0.15 (*P* < 0.001). Previous studies have reported similar findings, though with less pronounced differences [34–36]. Huang et al. [34] found pseudoprogression in 31% of cystic tumors versus 55% in solid tumors. In our cohort, pseudoprogression was observed in only 7% (*N* = 3) of microcystic tumors, compared to 44% (*N* = 14) of macrocystic and 31% (*N* = 68) of solid tumors. Huang et al. grouped macrocystic and microcystic tumors together, likely explaining the smaller observed differences [34–36]. Considering pseudoprogression in clinical decision-making, these findings suggest that microcystic tumors may be more favorable candidates for SRS. Although previous research has identified significant correlations between age, pretreatment growth rate, tumor size, and degree of MCP compression with increased treatment failure risk in long-term tumor control, these factors were not significantly associated with pseudoprogression in our study [15, 24, 37–39]. This supports the notion that pseudoprogression represents a separate phenomenon from long-term failure.

When comparing the dynamics of the solid versus the cystic components within tumors, a discordant behavior was mainly observed in the macrocystic tumors. In almost half (*N* = 19) of the 39 macrocystic tumors, the solid and cystic components exhibited opposite behavior; the solid components would increase while the cystic components would shrink, and vice versa. Four macrocystic tumors continued to grow despite regression of the solid tumor components, with three requiring additional interventions. In contrast, in microcystic tumors, the solid and cystic components generally behaved in a synchronized more predictable manner. This further supports the hypothesis that pseudoprogression occurrence after radiosurgery is related to the cellular composition of VS. Various hypotheses based on tumor biology have been described. Evidence indicates that microcystic intratumoral components in tissues correlate with a higher ratio of Antoni Type B tissue, which is generally more loosely organized, in contrast to the highly cellular and denser Antoni Type A tissue [40]. It could be hypothesized that more homogeneous tumors, with a higher Antoni Type A ratio, are more susceptible to pseudoprogression. Further research is required to unravel the underlying mechanisms of pseudoprogression following stereotactic radiosurgery.

This study has several limitations. The foremost is selection bias in the clinical decision-making process for managing Koos grade 4 VS with SRS, which affects several key patient characteristics in this cohort. For instance, the median and maximum Koos grade 4 tumor sizes for SRS are generally lower compared to the Koos grade 4 VS treated surgically [25, 41]. Due to inherent size constraints of SRS, the median tumor size in this cohort was 6.8 cm³, with a maximum of 19 cm³, indicating that giant Koos 4 VS are underrepresented. The selection bias also extends to the presenting symptomatology at treatment, with a relatively low incidence of mass effect symptoms and a relatively high rate of serviceable hearing.

Another significant limitation is the retrospective nature of this study, resulting in variability in follow-up regimens. Not all patients underwent early follow-up imaging at approximately 6 months post-GKRS, potentially influencing the time-to-peak and maximum volume increase values and resulting in up to a 13% (*N* = 33) underestimation of the true pseudoprogression incidence. Furthermore, secondary treatment within two years (*N* = 9) may introduce an additional underestimation in 3% of patients, since no data on tumor evolution post-intervention can be considered. For large Koos grade 4 tumors, this knowledge is practically infeasible to obtain fully in daily practice. Prompt intervention should be considered to mitigate the risk of mass effect complications when a significant volumetric increase is observed. Overall, pseudoprogression occurrence remained undetermined in 16% of the tumors, suggesting true pseudoprogression rate in this cohort ranges between 29% and 45%.

Finally, this study focused on temporal volumetric dynamics and did not account for the clinical consequences of pseudoprogression in terms of symptomatology. The clinical impact of pseudoprogression, including rates of new or worsened symptoms and the need for intervention, would also be valuable for clinical decision-making. However, in a retrospective cohort, reliably attributing clinical symptoms from medical records to volumetric changes is challenging, and extraction of such data would likely be incomplete and potentially introduce bias. To avoid overinterpretation, this study therefore deliberately focused on volumetric temporal dynamics and did not report these specific outcomes. However, a separate case-by-case evaluation of intervention indication, including the potential role of pseudoprogression following GKRS, was conducted to provide additional clinical context (Supplementary Results S5). Future prospective comparative matched-cohort studies of SRS versus microsurgery, incorporating standardized clinical outcome measures, are warranted. These studies are needed to improve assessment of the clinical impact of pseudoprogression and the role of conversion to microsurgery following SRS in optimizing individualized treatment selection for patients with Koos grade 4 VS.

Despite these limitations, this study provides valuable evidence on the temporal dynamics of pseudoprogression, such as time-to-peak and maximum relative increase, in Koos grade 4 VS post-GKRS. Given the associated surgical risks that significantly escalate with tumor size, these findings are particularly relevant to consider within the clinical decision-making for borderline SRS-eligible cases with a low symptomatic profile at the time of treatment or those relatively unsuitable for invasive surgery. During post-SRS follow-up it may aid in differentiating between tumors that are likely to fail and those that may regress, preventing unnecessary premature intervention and supporting a more individualized approach to SRS candidacy and follow-up in patients with Koos grade 4 VS.

## Conclusions

Pseudoprogression rates in selected Koos grade 4 VS appear comparable to rates of smaller tumors. Most cases peaked within two years, typically showing less than 50% volume increase at peak. Alternate patterns with later pseudoprogression may occur, supporting careful observation for up to 4 years in low-symptomatic patients to avoid unnecessary premature intervention. Traditional factors such as age, tumor size, degree of MCP compression, peritumoral edema, and pretreatment growth rate were not significantly associated with pseudoprogression. Tumors with microcystic components exhibited significantly lower pseudoprogression rates, potentially rendering them more suitable candidates for SRS. In contrast, macrocystic tumors demonstrated more variable dynamics, warranting careful monitoring in these tumors.

## Electronic Supplementary Material

Below is the link to the electronic supplementary material.

Supplementary Material 1

## Data Availability

The data that support the findings of this study are not publicly available due to restrictions applying to the availability of these data. Data are, however, available from the corresponding author upon reasonable request and are located in controlled access data storage at the Elisabeth Twee-Steden Hospital, Tilburg, The Netherlands.

## References

[1] Carlson ML, Link MJ (2021) Vestibular schwannomas. N Engl J Med 384:1335–1348. 10.1056/NEJMra2020394 33826821 10.1056/NEJMra2020394

[2] Goldbrunner R, Weller M, Regis J, Lund-Johansen M, Stavrinou P, Reuss D et al (2020) Eano guideline on the diagnosis and treatment of vestibular schwannoma. Neuro Oncol 22:31–45. 10.1093/neuonc/noz153 31504802 10.1093/neuonc/noz153 PMC6954440

[3] Tsao MN, Sahgal A, Xu W, De Salles A, Hayashi M, Levivier M et al (2017) Stereotactic radiosurgery for vestibular schwannoma: Int Ster Radio Soc (ISRS) Practice Guideline. 5 PMC5675503 29296459

[4] Bin-Alamer O, Osama M, Sheehan JP (2026) The evolution of stereotactic radiosurgery for vestibular schwannomas: recent evidence and outcomes. J Neurooncol 176. 10.1007/s11060-025-05252-1 10.1007/s11060-025-05252-1 PMC12521331 41085808

[5] Morshed RA, Arora T, Theodosopoulos PV (2021) Multimodality Treatment of Large Vestibular Schwannomas. Curr Otorhinolaryngol Rep 9:155–161. 10.1007/s40136-021-00336-8

[6] Starnoni D, Giammattei L, Cossu G, Link MJ, Roche PH, Chacko AG et al (2020) Surgical management for large vestibular schwannomas: a systematic review, meta-analysis, and consensus statement on behalf of the EANS skull base section. Acta Neurochir (Wien) 2:2595–2617. 10.1007/s00701-020-04491-7 10.1007/s00701-020-04491-7 PMC7550309 32728903

[7] Tuleasca C, Kotecha R, Sahgal A, de Salles A, Fariselli L, Paddick I et al (2023) Single-fraction radiosurgery outcomes for large vestibular schwannomas in the upfront or post-surgical setting: a systematic review and International Stereotactic Radiosurgery Society (ISRS) Practice Guidelines. J Neurooncol 165:1–20. 10.1007/s11060-023-04455-8 37843727 10.1007/s11060-023-04455-8 PMC10638172

[8] Dumot C, Pikis S, Mantziaris G, Xu Z, Anand RK, Nabeel AM et al (2022) Stereotactic radiosurgery for Koos grade IV vestibular schwannoma in young patients: a multi-institutional study. J Neurooncol 160:201–208. 10.1007/s11060-022-04134-0 36166113 10.1007/s11060-022-04134-0

[9] Umekawa M, Shinya Y, Hasegawa H, Kawashima M, Shin M, Katano A et al (2022) Stereotactic radiosurgery ensures an effective and safe long-term control of Koos grade IV vestibular schwannomas: a single-center, retrospective, cohort study. J Neurooncol. 10.1007/s11060-022-04058-9 35729368 10.1007/s11060-022-04058-9

[10] Breshears JD, Chang J, Molinaro AM, Sneed PK, McDermott MW, Tward A et al (2019) Temporal dynamics of pseudoprogression after gamma knife radiosurgery for vestibular schwannomas - A retrospective volumetric study. Clin Neurosurg 84:123–131. 10.1093/neuros/nyy019 10.1093/neuros/nyy019 29518221

[11] Fouard O, Daisne JF, Wanet M, Regnier M, Gustin T (2022) Long-term volumetric analysis of vestibular schwannomas following stereotactic radiotherapy: Practical implications for follow-up. Clin Transl Radiat Oncol 33:1–6. 10.1016/j.ctro.2021.12.003 34977365 10.1016/j.ctro.2021.12.003 PMC8688865

[12] Matsuo T, Okunaga T, Kamada K, Izumo T, Hayashi N, Nagata I (2015) Long-term follow-up results of linear accelerator-based radiosurgery for vestibular schwannoma using serial three-dimensional spoiled gradient-echo MRI. J Clin Neurosci 22:320–325. 10.1016/j.jocn.2014.06.100 25443082 10.1016/j.jocn.2014.06.100

[13] Wowra B, Muacevic A, Jess-Hempen A, Hempel JM, Müller-Schunk S, Tonn J (2005) Outpatient gamma knife surgery for vestibular schwannoma: definition of the therapeutic profile based on a 10-year experience. J Neurosurg 102:114–118. 10.3171/sup.2005.102.s_supplement.0114 15662792

[14] Nagano O, Serizawa T, Higuchi Y, Matsuda S, Sato M, Yamakami I et al (2010) Tumor shrinkage of vestibular schwannomas after Gamma Knife surgery: results after more than 5 years of follow-up. J Neurosurg 113:122–127. 10.3171/2010.8.GKS10960 21222292

[15] Van De Langenberg R, Hanssens PEJ, Verheul JB, Van Overbeeke JJ, Nelemans PJ, Dohmen AJC et al (2011) Management of large vestibular schwannoma. Part II. Primary Gamma Knife surgery: Radiological and clinical aspects - Clinical article. J Neurosurg 115:885–893. 10.3171/2011.6.JNS101963 21838503 10.3171/2011.6.JNS101963

[16] Hayhurst C, Zadeh G (2012) Tumor pseudoprogression following radiosurgery for vestibular schwannoma. Neuro Oncol 14:87–92. 10.1093/neuonc/nor171 22028389 10.1093/neuonc/nor171 PMC3245992

[17] Yu CP, Cheung JY, Leung S, Ho R Sequential volume mapping for confirmation of negative growth in vestibular schwannomas treated by gamma knife radiosurgery. J Neurosurg 2000:82–89. 10.3171/jns.2000.93 10.3171/jns.2000.93.supplement 11143269

[18] Hwang I, Choi SH, Kim JW, Yeon EK, Lee JY, Yoo RE et al (2022) Response prediction of vestibular schwannoma after gamma-knife radiosurgery using pretreatment dynamic contrast-enhanced MRI: a prospective study. Eur Radiol. 10.1007/s00330-021-08517-1 35084518 10.1007/s00330-021-08517-1

[19] Nakamura H, Jokura H, Takahashi K, Boku N, Akabane A, Yoshimoto T (2000) Serial follow-up MR imaging after gamma knife radiosurgery for vestibular schwannoma. AJNR Am J Neuroradiol 21:1540–1546 11003293 PMC7974039

[20] Kim Y-H, Kim DG, Han JH, Chung H-T, Kim IK, Song SW et al (2013) Hearing Outcomes After Stereotactic Radiosurgery for Unilateral Intracanalicular Vestibular Schwannomas: Implication of Transient Volume Expansion. Int J Radiat Oncol 85:61–67. 10.1016/j.ijrobp.2012.03.036 10.1016/j.ijrobp.2012.03.036 22580122

[21] Kim JH, Jung HH, Chang JH, Chang JW, Park YG, Chang WS (2017) Predictive Factors of Unfavorable Events After Gamma Knife Radiosurgery for Vestibular Schwannoma. World Neurosurg 107:175–184. 10.1016/j.wneu.2017.07.139 28826715 10.1016/j.wneu.2017.07.139

[22] Iorio-Morin C, Alsubaie F, Mathieu D (2016) Safety and efficacy of gamma knife radiosurgery for the management of Koos Grade 4 vestibular schwannomas. Neurosurgery 78:521–528. 10.1227/NEU.0000000000001154 26606668 10.1227/NEU.0000000000001154

[23] Piccirillo E, Wiet MR, Flanagan S, Dispenza F, Giannuzzi A, Mancini F et al (2009) Cyst Vestib Schwannoma Otol Neurotol 30:826–834. 10.1097/MAO.0b013e3181b04e18 10.1097/MAO.0b013e3181b04e18 19704364

[24] Hasegawa T, Kato T, Naito T, Tanei T, Ishii K, Tsukamoto E et al (2021) Predictors of long-term tumor control after stereotactic radiosurgery for Koos grade 4 vestibular schwannomas. J Neurooncol 151:145–156. 10.1007/s11060-020-03622-5 33415658 10.1007/s11060-020-03622-5

[25] Pikis S, Mantziaris G, Kormath Anand R, Nabeel AM, Sheehan D, Sheehan K et al Stereotactic radiosurgery for Koos grade IV vestibular schwannoma: a multi-institutional study. J Neurosurg 2022:1–8. 10.3171/2022.4.jns22203 10.3171/2022.4.JNS22203 36303474

[26] Varughese JK, Breivik CN, Wentzel-Larsen T, Lund-Johansen M (2012) Growth of untreated vestibular schwannoma: A prospective study - Clinical article. J Neurosurg 116:706–712. 10.3171/2011.12.JNS111662 22264178 10.3171/2011.12.JNS111662

[27] Cornelissen S, Schouten SM, Langenhuizen PPJH, Lie S, Te, Kunst HPM, de With PHN et al defining tumor growth in vestibular schwannomas: a volumetric inter-observer variability study in contrast-enhanced T1-weighted MRI. Neuroradiology 2024:2024.03.15.24304080. 10.1101/2024.03.15.24304080 10.1007/s00234-024-03416-w PMC11534841 38980343

[28] Balossier A, Olteanu M, Delsanti C, Troude L, Thomassin JM, Roche PH et al (2025) Dynamics of tumor evolution after Gamma Knife radiosurgery for sporadic vestibular schwannoma: Defining volumetric patterns characterizing individual trajectory. Neuro Oncol 27:545–556. 10.1093/neuonc/noae187 39283980 10.1093/neuonc/noae187 PMC11812029

[29] Rueß D, Schütz B, Celik E, Baues C, Jünger ST, Neuschmelting V et al (2023) Pseudoprogression of Vestibular Schwannoma after Stereotactic Radiosurgery with Cyberknife^®^: Proposal for New Response Criteria. Cancers (Basel) 15:1496. 10.3390/cancers15051496 36900290 10.3390/cancers15051496 PMC10000564

[30] Nagano O, Higuchi Y, Serizawa T, Ono J, Matsuda S, Yamakami I et al (2008) Transient expansion of vestibular schwannoma following stereotactic radiosurgery: Clinical article. J Neurosurg 109:811–816. 10.3171/JNS/2008/109/11/0811 18976069 10.3171/JNS/2008/109/11/0811

[31] Langenhuizen PPJH, Sebregts SHP, Zinger S, Leenstra S, Verheul JB, de With PHN (2020) Prediction of transient tumor enlargement using MRI tumor texture after radiosurgery on vestibular schwannoma. Med Phys 47:1692–1701. 10.1002/mp.14042 31975523 10.1002/mp.14042 PMC7217023

[32] Rueß D, Pöhlmann L, Hellerbach A, Hamisch C, Hoevels M, Treuer H et al (2018) Acoustic Neuroma Treated with Stereotactic Radiosurgery: Follow-up of 335 Patients. World Neurosurg 116:e194–202. 29715569 10.1016/j.wneu.2018.04.149

[33] Pollock BE (2006) Management of vestibular schwannomas that enlarge after stereotactic radiosurgery: Treatment recommendations based on a 15 year experience. Neurosurgery 58:241–246. 10.1227/01.NEU.0000194833.66593.8B 16462477 10.1227/01.NEU.0000194833.66593.8B

[34] Huang C-Y, Peng S-J, Yang H-C, Wu H-M, Chen C-J, Wang M-C et al (2023) Association Between Pseudoprogression of Vestibular Schwannoma After Radiosurgery and Radiological Features of Solid and Cystic Components. Neurosurgery 93:1383–1392. 10.1227/neu.0000000000002599 37432016 10.1227/neu.0000000000002599

[35] Bowden G, Cavaleri J, Monaco E, Niranjan A, Flickinger J, Lunsford LD (2017) Cystic vestibular schwannomas respond best to radiosurgery. Neurosurgery 81:490–497. 10.1093/neuros/nyx027 28368501 10.1093/neuros/nyx027

[36] Wu CC, Guo WY, Chung WY, Wu HM, Lin CJ, Lee CC et al (2017) Magnetic resonance imaging characteristics and the prediction of outcome of vestibular schwannomas following Gamma Knife radiosurgery. J Neurosurg 127:1384–1391. 10.3171/2016.9.JNS161510 28186452 10.3171/2016.9.JNS161510

[37] Langenhuizen PPJH, Zinger S, Hanssens PEJ, Kunst HPM, Mulder JJS, Leenstra S et al (2019) Influence of pretreatment growth rate on Gamma Knife treatment response for vestibular schwannoma: A volumetric analysis. J Neurosurg 131:1405–1412. 10.3171/2018.6.JNS18516 30497177 10.3171/2018.6.JNS18516

[38] Shah F, Hamilton LOW, Yiannakis CP, Mohd Slim MA, Kontorinis G A systematic review and meta-Analysis of Stereotactic radiosurgery as primary treatment in fast-growing vestibular schwannomas. J Laryngol Otol 2023:15–19. 10.1017/S0022215123000786 10.1017/S0022215123000786 37194631

[39] Huang C-W, Tu H-T, Chuang C-Y, Chang C-S, Chou H-H, Lee M-T et al (2018) Gamma Knife radiosurgery for large vestibular schwannomas greater than 3 cm in diameter. J Neurosurg 128:1380–1387. 10.3171/2016.12.JNS161530 28707997 10.3171/2016.12.JNS161530

[40] Wippold FJ, Lubner M, Perrin RJ, Lämmle M, Perry A (2007) Neuropathology for the neuroradiologist: Antoni A and Antoni B tissue patterns. Am J Neuroradiol 28:1633–1638. 10.3174/ajnr.A0682 17893219 10.3174/ajnr.A0682 PMC8134199

[41] Schouten SM, Cornelissen S, Langenhuizen PPHJ, Jansen TTG, Mulder JJS, Derks J et al (2024) Wait-and-scan management in sporadic Koos grade 4 vestibular schwannomas: A longitudinal volumetric study. Neuro-Oncology Adv 6:vdad144. 10.1093/noajnl/vdad144 10.1093/noajnl/vdad144 PMC10771273 38187870

