# Supplementary material for: Pseudoprogression in Koos grade 4 vestibular schwannomas following stereotactic radiosurgery: temporal dynamics and radiological predictors
Source: J Neurooncol. 2026 May 25;178(1):19. doi: 10.1007/s11060-026-05619-y (PMC13201310; doi:10.1007/s11060-026-05619-y)
Supplement: Supplementary file 1 — Supplementary Material 1 [file 11060_2026_5619_MOESM1_ESM.pdf]

Supplementary Material

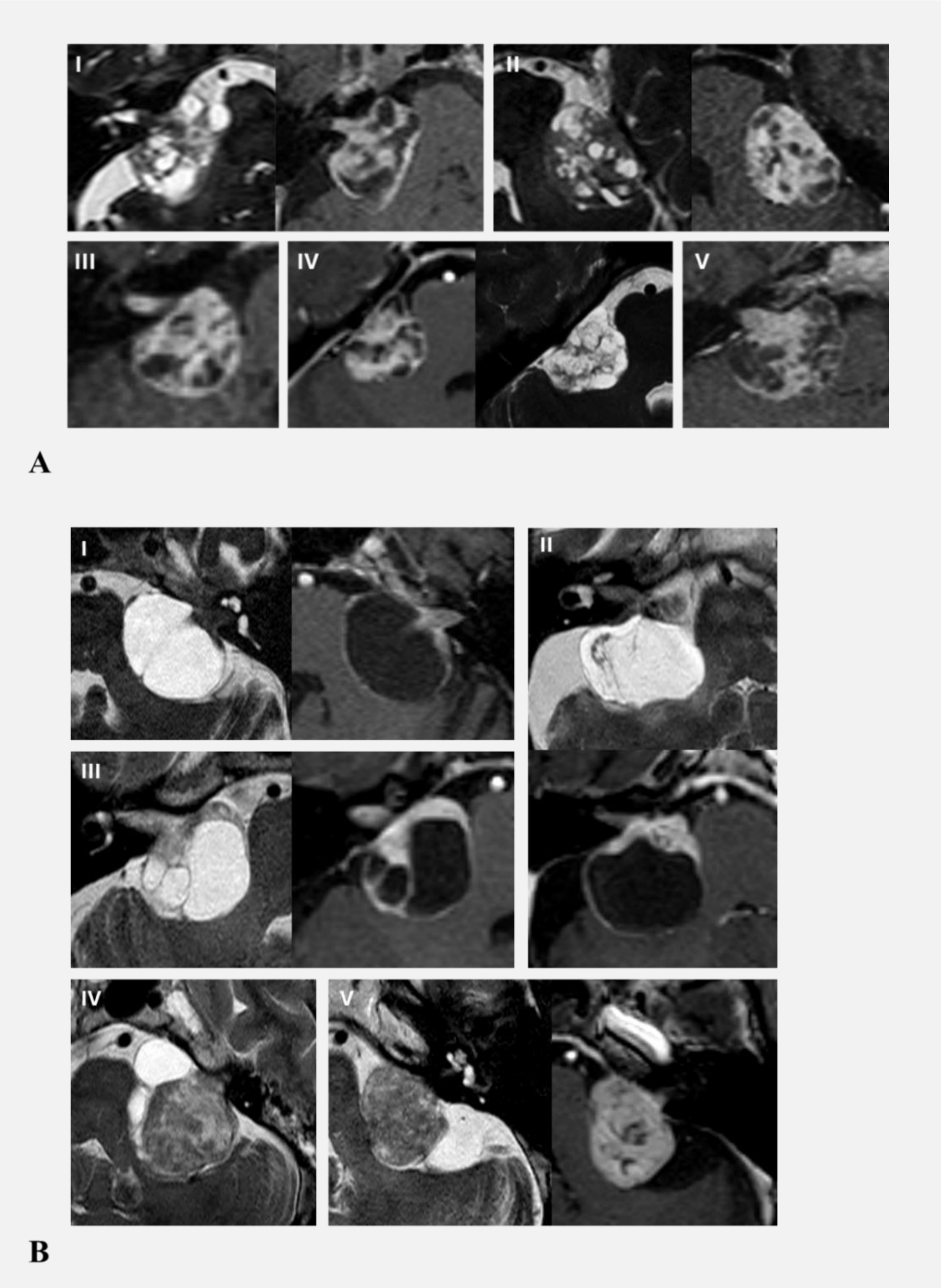

Supplementary Figure S1. Examples of tumors with microcystic components versus tumors with macrocystic components. (A) Five examples of tumors with microcystic components. (B) Five examples of tumors with macrocystic components, of which example I-III with intratumoral macrocysts and example IV and V with peritumoral macrocysts

S.M. Schouten, S. Cornelissen, P. Langenhuizen, H.P.M. Kunst, J.B. Verheul. Pseudoprogression in Koos grade 4 vestibular schwannomas following stereotactic radiosurgery: temporal dynamics and radiological predictors. Journal of Neuro-Oncology 2026. **Corresponding Author:** Sammy M. Schouten. Email: s.schouten@etz.nl

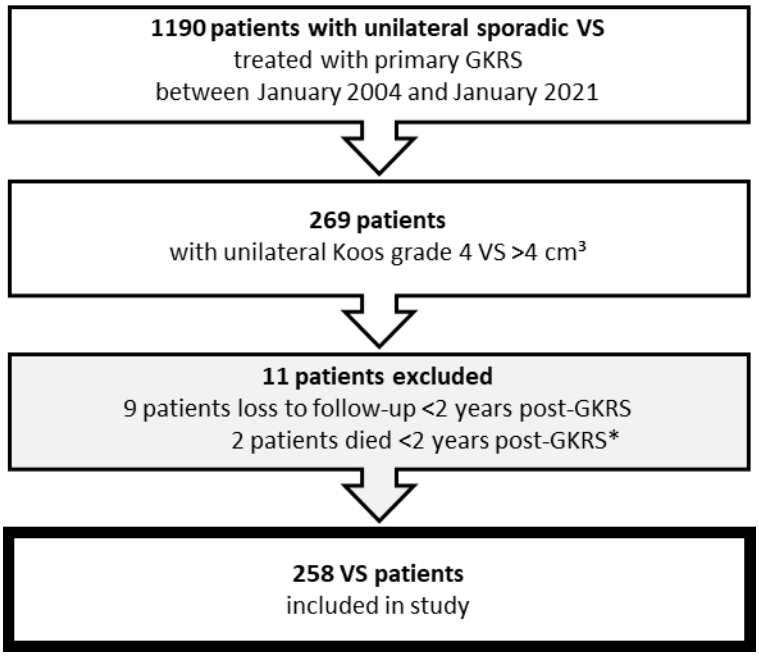

Supplementary Figure S2. Flow diagram study population. *Death was VS-unrelated. Abbreviation: VS, vestibular schwannoma. GKRS, Gamma Knife Radiosurgery.

 Corresponding Author: Sammy M. Schouten. Email: s.schouten@etz.nl

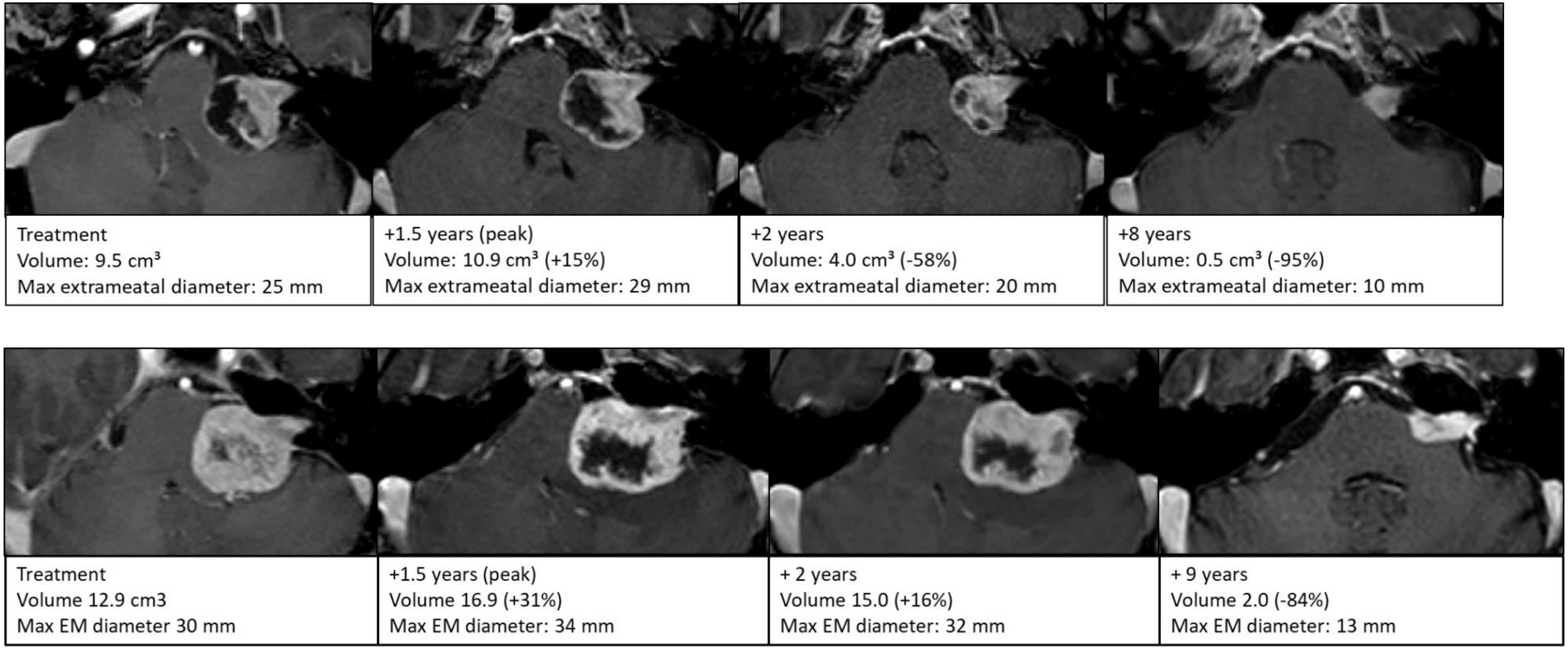

Supplementary Figure S3. Two examples of tumors with pseudoprogression from time of treatment and during follow-up.

S.M. Schouten, S. Cornelissen, P. Langenhuizen, H.P.M. Kunst, J.B. Verheul. Pseudoprogression in Koos grade 4 vestibular schwannomas following stereotactic radiosurgery: temporal dynamics and radiological predictors. Journal of Neuro-Oncology 2026. **Corresponding Author:** Sammy M. Schouten. Email: s.schouten@etz.nl

Supplementary Results S4. Sensitivity analyses missing pseudoprogression data.

1. **Undetermined cases for pseudoprogression and impact on rates**

   Undetermined due to:
   1. Continuous regression without an available follow-up scan at 3-9 months post-GKRS
   2. Subsequent early intervention within two years post-GKRS following observed enlargement

Presence of pseudoprogression N (% of total cohort)				
**YES**	**NO**	**UNKNOWN Missing early scan**	**UNKNOWN Early intervention**	**Total cohort**
75 (29%)	141 (55%)	33 (13%)	9 (3%)	258 (100%)

Pseudoprogression rates based on different assumptions:

Assumption	Pseudoprogression rate in %
1. Unknown cases classified as NO pseudoprogression (total N=258)	29%
2. Unknown cases classified as YES pseudoprogression (total N=258)	45%
3. Unknown cases missing early scan classified as NO pseudoprogression Excluding unknown due to early intervention (total N=249)	30%
4. Unknown cases missing early scan classified as YES pseudoprogression Excluding unknown due to early intervention (total N=249)	43%
5. Unknown due to early intervention classified as NO pseudoprogression<br>6. Excluding unknown due to missing early scan (total N=225)	33%
7. Unknown missing early scan classified as NO pseudoprogression<br>8. Excluding unknown due to missing early scan (total N=225)	35%

2. **Availability scan intervals for pseudoprogression tumors (N=75) at 3-9 months, 12 months, and 24 months post-SRS**

	**3-9 months post-GKRS**	**12 months Post-GKRS**	**24 months Post-GKRS**
N (% of pseudoprogression) missing follow-up	N=14 (19%)	N=3	N=0
**Time to peak data in missing cases**<br>Impact on time to peak data	**Peak at 2 months: N=1**<br>Peak could be later and/or higher<br><br>**Peak at 1 year: N=7**<br>Peak could be earlier and/or higher<br><br>**Peak >2 years: N=6**<br>No impact on peak data	**Peak at 6 months: N=2**<br>Peak could be later and/or higher<br><br>**Peak at 2 years: N=1**<br>Peak could be earlier and/or higher	N/A
Missing other key scan intervals of missing scans	0/14 (0%) have missing scans at 1 and 2 years follow-up	0/3 (0%) have missing scans at 3-9 months or 24 months follow-up	N/A

S.M. Schouten, S. Cornelissen, P. Langenhuizen, H.P.M. Kunst, J.B. Verheul. Pseudoprogression in Koos grade 4 vestibular schwannomas following stereotactic radiosurgery: temporal dynamics and radiological predictors. Journal of Neuro-Oncology 2026. **Corresponding Author:** Sammy M. Schouten. Email: s.schouten@etz.nl

3. Multivariable logistic analyses: summary comparison of different classification assumptions of the undetermined cases for pseudoprogression, including the adjusted odds ratios (aOR), CI95%, and overall Wald *P*-values:

a) Assumption "all underdetermined cases" included:
- ***Classified as YES pseudoprogression***
  Microcystic still significantly inversely correlated in multivariable analyses with pseudoprogression aOR 0.19 (CI95% 0.09-0.44 overall Wald $P < 0.001$)
  Still no other significant factors in multivariable analyses
- ***Classified as NO pseudoprogression***
  Microcystic still significantly inversely correlated in multivariable analyses with pseudoprogression aOR 0.16 (CI95% 0.07-0.48 overall Wald $P = 0.002$)
  Still no other significant factors in multivariable analyses

b) Assumption "undetermined cases due to treatment < 2 years" included
- ***Classified as NO pseudoprogression***
  Microcystic still significantly inversely correlated in multivariable analyses with pseudoprogression aOR 0.14 (CI95% 0.05-0.41, overall Wald $P = 0.001$)
  Still no other significant factors in multivariable analyses
- ***Classified as YES pseudoprogression***
  Microcystic still significantly inversely correlated in multivariable analyses with pseudoprogression aOR 0.12 (CI95% 0.04-0.35, overall Wald $P < 0.001$)
  Still no other significant factors in multivariable analyses

c) Assumption "undetermined cases due to missing early scan" included
- ***Classified as YES pseudoprogression***
  Microcystic still significantly inversely correlated in multivariable analyses with pseudoprogression aOR 0.21 (CI95% 0.09-0.47, overall Wald $P < 0.001$)
  Still no other significant factors in multivariable analyses
- ***Classified as NO pseudoprogression***
  Microcystic still significantly inversely correlated in multivariable analyses with pseudoprogression aOR 0.16 (CI95% 0.05-0.47, overall Wald $P < 0.001$)
  Still no other significant factors in multivariable analyses

Supplementary Results S5. Additional intervention data.

Patient baseline characteristics				Pseudoprogression		Additional treatment indication factors							
ID	Age in years	Tumor volume in cm3	Cystic components	Presence	Reason unknown	Intervention	Time in months	Tumor progression	Mass effect symptoms	Intervention pseudoprogression-related?	In retrospect conversion premature?	Indication notes (% compared to treatment volume at second intervention)
1	45	4.4	macro	Unknown	Treatment <2 years	Surgery	3	**Yes**	**Yes**	**Maybe**	No	Progression macrocyst (173%) & development facial nerve palsy HB 2-3
2	71	10.6	macro	Unknown	Treatment <2 years	Surgery	3	**Yes**	**Yes**	**Maybe**	No	Progression macrocyst (50%) & already cerebellar symptoms at time of treatment
3	82	14.6	macro	Unknown	Treatment <2 years	Surgery	4	**Yes**	**Yes**	**Maybe**	No	Progression macrocyst (45%) & already cerebellar symptoms at time of treatment
4	67	6.8	none	Yes		Shunt	5	No	**Yes**	No	No	No progression (-71%) & development normal pressure hydrocephalus
5	70	4.4	none	Yes		Shunt	8	No	**Yes**	No	No	No progression (-40%) & development normal pressure hydrocephalus
6	63	10.0	none	Yes		Shunt	12	No	**Yes**	No	No	No progression (5%) & development normal pressure hydrocephalus
7	41	5.4	none	Unknown	Treatment <2 years	Surgery	20	**Yes**	No	**Maybe**	**Maybe**	Continued progression (73%)
8	64	13.8	macro	Yes		Surgery	20	**Yes**	**Yes**	No	No	Progression macrocyst & development cerebellar symptoms
9	43	5.3	none	Yes		Surgery	22	No	No	No	No	Stable tumor & pre-existent persistent trigeminal neuralgia.
10	56	6.9	none	Unknown	No 6 months follow-up	Surgery	28	**Yes**	No	**Maybe**	**Maybe**	Continued progression (55%)
11	51	5.7	none	No		Second GKRS	29	**Yes**	No	No	No	Initial shrinkage to -30% then progression (5%)
12	51	11.5	none	No		Shunt & surgery	31	**Yes**	**Yes**	No	No	Continued progression (51%) & development hydrocephalus
13	65	5.5	none	Unknown	No 6 months follow-up	Second GKRS	31	**Yes**	No	**Maybe**	**Maybe**	Continued progression (66%)
14	52	6.7	none	Yes		Shunt	32	**Yes**	**Yes**	**Yes**	No	Pseudoprogression peak at 3.2 years (64%) & development hydrocephalus
15	75	4.6	none	Unknown	No 6 months follow-up	Second GKRS	33	**Yes**	No	No	No	Initial shrinkage to -33% then continued progression (47%)
16	70	13.7	macro	No		Shunt	37	**Yes**	No	No	No	Regressing solid component, progression macrocyst (-11%)
17	44	5.9	none	Unknown	No 6 months follow-up	Surgery	37	**Yes**	**Yes**	No	No	Continued growth (62%) & trigeminal hyperesthesia
18	71	18.7	none	No		Second GKRS	37	**Yes**	No	No	No	Initial shrinkage to -23 then progression (15%)
19	37	7.0	none	No		Surgery	38	**Yes**	**Yes**	No	No	Continuous progression (60%) & development mild trigeminal symptoms
20	55	6.4	none	No		Surgery	38	**Yes**	No	No	No	Continuous progression (77%)
21	43	8.7	none	No		Surgery	41	**Yes**	No	No	No	Initial shrinkage to -68% then progression (4%)
22	42	4.7	micro	No		Second GKRS	49	**Yes**	No	No	No	Initial shrinkage to -71% then progression (2%)
23	48	10.3	none	Unknown	No 6 months follow-up	Surgery	51	**Yes**	No	No	No	Initial shrinkage -35% then progression (15%)
24	73	4.2	none	No		Second GKRS	62	**Yes**	No	No	No	Initial shrinkage to -61% then progression (11%)
25	46	7.9	none	Yes		Second GKRS	65	**Yes**	No	No	No	Pseudoprogression, shrinkage to -52% then progression (-35%)
26	58	7.2	none	Yes		Second GKRS	80	**Yes**	No	No	No	Pseudoprogression, shrinkage to -81% then progression (-69%)
27	39	4.7	none	Unknown	No 6 months follow-up	Second GKRS	82	**Yes**	No	No	No	Initial shrinkage -51% to then progression (-4%)
28	56	6.8	none	No		Second GKRS	86	**Yes**	No	No	No	Initial shrinkage to -75% then progression (-50%)
29	77	4.8	none	No		Second GKRS	95	**Yes**	No	No	No	Initial shrinkage to -53% then progression (-11%)
30	71	5.4	none	No		Second GKRS	99	**Yes**	No	No	No	Initial shrinkage to -30% then oscillating progression (20%)
								**Total N=26**	**Total N=11**	**Total N=7**	**Total N=3**	

S.M. Schouten, S. Cornelissen, P. Langenhuizen, H.P.M. Kunst, J.B. Verheul. Pseudoprogression in Koos grade 4 vestibular schwannomas following stereotactic radiosurgery: temporal dynamics and radiological predictors. Journal of Neuro-Oncology 2026. **Corresponding Author:** Sammy M. Schouten. Email: s.schouten@etz.nl
